# Relationships between job satisfaction, organizational commitment, burnout and job performance of healthcare professionals in a district-level health care system of Shenzhen, China

**DOI:** 10.3389/fpsyg.2022.992258

**Published:** 2022-11-28

**Authors:** Xin Wang, Chaofan Li, Yuanqing Chen, Caiyun Zheng, Fei Zhang, Yixiang Huang, Stephen Birch

**Affiliations:** ^1^School of Public Health, Sun Yat-sen University, Guangzhou, China; ^2^Centre for Health Management and Policy Research, School of Public Health, Cheeloo College of Medicine, Shandong University, Jinan, China; ^3^NHC Key Lab of Health Economics and Policy Research, Shandong University, Jinan, China; ^4^Shenzhen Nanshan Medical Group, Shenzhen, China; ^5^Centre for the Business and Economics of Health, The University of Queensland, Brisbane, QLD, Australia

**Keywords:** healthcare professional, job satisfaction, job performance, organizational commitment, burnout

## Abstract

**Background:**

Existing research indicates that job satisfaction has effects on job performance, but little evidence exists about the mechanism through which the satisfaction-performance association operates. This study aims to examine the effect of job satisfaction on job performance in a district-level health care system of China and to explore the effect mechanism mediated by organizational commitment and burnout.

**Methods:**

Cluster sampling was used in this study. All healthcare professionals in the Nanshan Medical Group, who were at work in the last 3 months and able to complete online questionnaire independently were invited to participate the anonymous online survey. Job satisfaction, organizational commitment, burnout and job performance were measured by tools, which have been validated in China. Descriptive statistics were used for the socio-demographic variables and the four job psychological variables. Pearson correlation coefficients was used to determine associations among each of the psychological variables. Linear regression was used to examine association between job performance and other three psychological variables. PROCESS macro was used to examine the mediation effects of organizational commitment and burnout on the association between job satisfaction and performance.

**Results:**

In total, 1,200 healthcare professionals completed the anonymous online survey. Job satisfaction, organizational commitment, and job performance were positively correlated with one another, with burnout negatively correlated with them. Linear regression revealed that demographic characteristics, job satisfaction, organizational commitment, and burnout explained 5, 6, 2, and 9% of the variance in job performance. Path analysis showed that the coefficient of the direct effect of job satisfaction on job performance was 0.18, the coefficients of the indirect effects of job satisfaction on job performance through organizational commitment and burnout were 0.17 and 0.37, respectively. The coefficients of the indirect effects of organizational commitment on job performance through burnout was −0.04, but it was not significant.

**Conclusion:**

It is promising to improve job performance of providers in Chinese healthcare systems by improving job satisfaction and reducing burnout. Tailored support policies for female healthcare professionals, appropriate incentive mechanisms and improving multidisciplinary healthcare delivery are potential to improve job performance of healthcare professionals in integrated healthcare systems.

## Introduction

Job satisfaction of healthcare professionals is a significant concern in health systems (World Health Organization, 2020). It has been added to the Triple Aim for optimizing performance of health systems, and is now the fourth element of the Quadruple Aim (Lacagnina, 2018; Bachynsky, 2020). Job satisfaction is defined as the attitudes towards work and the related emotions, beliefs, and behaviors (Lu et al., 2005). Job satisfaction of healthcare professionals depends on both individual and organizational factors, such as personality, workload, remuneration, work environment and leadership style (Blaauw et al., 2013; Salvatore et al., 2018). Low job satisfaction of healthcare professionals has been shown to lead to low patient satisfaction, low quality of health care, low job performance, bad health outcomes, and might also have effects on well-being of healthcare professionals (Wang et al., 2017; Salvatore et al., 2018). Therefore, improving job satisfaction of healthcare professionals has been a health system strengthening initiative in many low- and middle-income countries besides increasing supply and improving quality of healthcare professionals.

Job satisfaction of healthcare professionals is generally low in China, especially among workers in primary care settings. A systematic review of thirteen studies showed that there was a decline in job satisfaction among urban primary health care professionals following health reforms in 2009 (Li et al., 2017). A cross-sectional study of 225 physicians in 2020 found that only 36.1% were satisfied or very satisfied with their job (Ma et al., 2021). In addition to job satisfaction, results from the previous national survey revealed considerable prevalence of exhaustion (41%), depersonalization (37%) and lacking personal accomplishment (34%) among healthcare professionals in China. The rates of prevalence were found to be higher among the low-satisfaction healthcare professionals than among the high-satisfaction healthcare professionals. Job satisfaction and related job psychology of healthcare professionals is attracting increasing attention among policy makers and managers.

According to the job demands-resources model, job demands such as high workload, interpersonal conflicts, and unfavorable work environment may lead to dissatisfaction and burnout, and negatively impact upon employees’ performance (Bakker and Demerouti, 2007). There has been a substantial amount of research on the relationship between job satisfaction and job performance in industrial-organizational psychology and human resource management (Ren and Zhong, 2022; Susanto et al., 2022). It consistently shows job satisfaction affecting job performance of healthcare professionals significantly, in terms of efficiency, quality of care and patient satisfaction (Sakdipath and Pongsatean, 2001; Chao et al., 2015; Hou et al., 2020). However, no clear picture emerged on the mechanism by which job satisfaction improves job performance. Healthcare professionals’ commitment to organization and their job burnout were the most widely independently or jointly identified mediate factors between healthcare professionals’ job satisfaction and performance. According to ICD-11, burnout is characterized as a syndrome, defined by three dimensions-exhaustion, reduced professional efficacy and increased mental distance from one’s job (Editorial, 2019). Social Identity Theory proposed that a more salient an organization is to a person’s identity, the more likely the person is to modify his or her behavior to align with organization norms, values and goals (Hogg, 2006). Therefore, organizational commitment and job burnout are supposed to have effect on job performance theoretically. Results from structural equation modeling in China found a significant direct effect of job satisfaction on burnout and job performance, and a significant indirect effect of job satisfaction on job performance through burnout as a mediator (Wang et al., 2020). A cross-sectional study of primary healthcare professionals in China showed that the effect of job satisfaction on job performance was mediated by organizational commitment (Zhao, 2015). Additionally, relationships have been found between job satisfaction, organizational commitment, job burnout and job performance. A study of Nigeria’s public healthcare employees found that job satisfaction was positively correlated with the commitment of healthcare workers to their organizations (Adeniji et al., 2019). Regression analysis by Majd et al. revealed that high career commitment among hospital based nurses was related to low burnout (Mrayyan and Al-Faouri, 2008). Correlations between organizational commitment and job performance showed the presence of a significant and positive relationship (Abdullah et al., 2021; Wayoi et al., 2021). Peng et al. explored the effect that organizational commitment had on job performance and the mediator role of burnout (Lu et al., 2020). Based on the findings of these studies, further development of a comprehensive model is required to test the pathway from job satisfaction to job performance.

This study aims to analyze job satisfaction, organizational commitment, burnout and job performance of healthcare professionals a healthcare system in China, and examine the mediation effects of organizational commitment and burnout on the association between job satisfaction and performance, providing evidence for policy makers concerned with performance improvement of healthcare professionals and healthcare institutions. With the development of integrated health care system worldwide, cross-institution management, especially human resource management, is getting more and more important. Findings of this study might provide evidence for cross-institution human resource management for other integrated health care systems worldwide.

## Materials and methods

### Study setting and design

This is a cross-sectional study conducted in Nanshan district, Shenzhen city in August 2020. The economy of Nanshan district has been ranked first among all 2,846 districts/counties of China since 2014. As one of the four largest cities in China, Shenzhen city is known for its fast development pace, high work pressure, and high cost of living. On a *per capita* basis, human and non-human resources for health care are much higher than the national average. Similarly, Shenzhen residents have higher demands of health care than people in other areas. To meet demands for comprehensive and high-quality health care, Nanshan district established a medical group with the aim of promoting health care integration in 2017. The group includes the Center for Disease Control and Prevention of Nanshan district (CDC), five district-level hospitals and 79 community health stations (CHSs). Following a three-year reform period for organizational and system integration, the Nanshan Medical Group turned its attention to integrated personnel management in order to improve job satisfaction and enthusiasm of all staff. Two of the authors are managers of the Nanshan Medical Group. This study is a baseline evaluation of job satisfaction and self-reported job performance of all healthcare professionals in the medical group, to provide evidence for policy making on personnel management.

### Participants and sampling

To ensure the representative of the sample, cluster sampling was used in this study. All healthcare professionals in the Nanshan Medical Group, who were at work in the last 3 months and able to complete online questionnaire independently were invited to participate the anonymous online survey. In regard to professions, it includes health educators, general practitioners, specialist physicians, nurses, public health physicians, pharmacists and so on. Participation in the survey was voluntary with a consent form, and the participants were not compensated. This study was approved by the Ethics Committee of School of Public Health, Sun Yat-sen University.

### Measures and variables

#### Job performance

We measure self-reported job performance using an instrument developed by Zhao et al. (2015b) in Chinese primary care setting, which has shown good reliability and validity in previous studies. The 12-item instrument is categorized into three dimensions, task performance (4 items), interpersonal performance (5 items) and contextual performance (3 items). Cronbach’s α for the three dimensions were 0.805, 0.714, and 0.842. Each item was scored on a seven-point Likert scale, ranging from strongly disagree (0) to 6 (strongly agree). The total possible score ranges from 0 to 72, with a higher total score indicating a higher perceived job performance. In the present study, the Cronbach’s α for the whole instrument was 0.954.

#### Job satisfaction

We assessed job satisfaction with a tool which was developed based on the existing literature and measurement instruments. The tool was validated with high-level reliability and validity in Chinese primary care settings (Zhang and Feng, 2011; Wang et al., 2020). It consists of eighteen items in five dimensions, including 4 items about satisfaction of job characteristics, 3 items about satisfaction of practicing environment, 3 items about satisfaction of job rewards, 3 items about satisfaction of interpersonal relationship, and 5 items about satisfaction of institutional management. The internal consistency coefficients of the above five dimensions were 0.832, 0.809, 0.813, 0.832, and 0.844. Participants were asked to rate each item according to their daily work experience. The responses were measured on a 5-point Likert scale form 1, very dissatisfied to 5, very satisfied. The total possible score ranges from 18 to 90, with a higher total score indicating a higher job satisfaction. The Cronbach’s α for the job satisfaction instrument in this study was 0.850.

#### Organizational commitment

The commitment of healthcare professionals to their organizations was measured using a scale developed by Lin et al. in China which has established validity (Ling et al., 2001; Zhao, 2015; Adeniji et al., 2019; Cao et al., 2019). The Organizational Commitment Scale consists of 25 items categorized into five dimensions, economic commitment, affective commitment, ideal commitment, opportunity commitment, and normative commitment. Cronbach’s α for each dimension of the scale were 0.708, 0.760, 0.763, 0.784, and 0.799. Each dimension includes five items, and all items were rated on a 4-point Likert scale ranging from 1 (strongly disagree) to 4 (strongly agree). The total possible score ranges from 25 to 100, with a higher total score indicating a higher organizational commitment. The Cronbach’s α for the Organizational Commitment Scale in this study was 0.919.

#### Job burnout

Job burnout is characterized by a state of mental exhaustion, depersonalization, and a decreased sense of personal accomplishment. In the present study, it was assessed by the Maslach Burnout Inventory General Survey with 22 items (Zhang, 2011). MBI-GS includes emotional exhaustion (9 items), depersonalization (5 items), and reduced personal accomplishment (8 items). The internal consistency coefficients of the above three dimensions were 0.842, 0.819, and 0.826. The response options for each item on the MBI-GS are rated on a seven-point Likert scale, from never having those feelings (0) to having those feelings everyday (Salvatore et al., 2018). The total possible score ranges from 0 to 132, with a higher total score indicating a higher occupational burnout. The Cronbach’s α for the MBI-GS in this study was 0.805.

### Data collection

An online questionnaire was developed to collect data on social-demographic information, scales of job satisfaction, organizational commitment, burnout, and job performance. Data were collected by using an online data collection tool SO JUMP (China Webmaster, 2006). With the coordination of Nanshan Medical Group, a link to an informed consent and the online questionnaire were sent to all potential participants’ mailboxes through an automatic office system in August 2020. Once the questionnaire was completed and submitted, it was resubmitted to SO JUMP. After logging in, researchers (XW and CL) could download the original data immediately. Managers of Nanshan Medical Group did not participate in data collection.

### Data analysis

First, descriptive statistics was conducted for all the categorical demographic and social-economic variables to obtain the basic characteristics of respondents, including frequency (n) and percentage (%). Second, we calculated mean and standard deviation for the four continuous variables, including job satisfaction, organizational commitment, burnout and self-reported job performance. Third, t test and one-way ANOVA were used to examine difference in job performance between subgroups of demographic and social-economic characteristics. Forth, Pearson correlation coefficients were used to determine associations among each of the psychological variables without controlling for demographic and social-economic variables. Fifth, linear regression analysis was used to examine associations between job performance and the other three psychological variables, adjusting for demographic and social-economic variables. Last, PROCESS macro introduced by Hayes (2018) was used to examine the mediation effects of organizational commitment and burnout on the association between job satisfaction and performance. The reasons for choosing PROCESS, rather than structural equation modelling to test the mediation hypothesis were: (1) controlling for demographic and social-economic variables when examining the association between psychological variables, and (2) obtaining bootstrap confidence interval for the mediation analysis. The descriptive statistics, *t* test, ANOVA, correlation analyses and linear regressions were performed using Stata 15.1 and PEOCESS macro for SPSS was used to test mediation relationship. Statistical significance was set at *p* < 0.05.

## Results

### Participants’ characteristics and descriptive statistics

As shown in Table 1, a total of 1,200 participants (total response 67.26%) completed the online survey, with average job performance score of 65.52. Approximately three quarters of participants were female, and two thirds were under 40-years old. Most of the participants received three-year education in college or undergraduate education in university (87.41%), with primary or intermediate professional titles (82.83%). Half of the participants (53.1%) have been working for less than 5 years, and majority of them (91.7%) earned more than ¥5,000 per month. Most of participants were from county-level hospitals (43.60%) and CHS (50.60%). The male, elderly, participants with high professional title, long working years and high income had higher job performance than other participants. There were no significant differences in job performance across subgroups of educational level and institution.

**Table 1:** Respondent characteristics.

Variables	*N* (%)	Job performance, Mean (SD)	*p*
Gender			
Male	276 (23.00)	67.24 (10.66)	0.002
Female	924 (77.00)	65.00 (10.66)	
Age (years old)			
<30	276 (23.00)	63.10 (10.59)	<0.001
30–39	500 (41.70)	65.46 (10.11)	
40–49	216 (18.00)	67.87 (11.10)	
≥50	72 (6.00)	70.04 (11.10)	
Education level			
Three-Year College or below	217 (18.10)	64.49 (10.58)	0.11
Bachelor	832 (69.30)	65.95 (10.83)	
Master and above	151 (12.60)	64.60 (10.01)	
Professional title			
No title	102 (8.50)	63.42 (11.18)	<0.001
Primary	474 (39.50)	64.20 (9.92)	
Intermediate	520 (43.30)	66.36 (10.55)	
Vice-senior/senior	104 (8.70)	69.35 (12.84)	
Years of working			
≤5	637 (53.10)	64.35 (10.05)	<0.001
6–10	247 (20.60)	65.17 (11.93)	
11–20	256 (21.30)	67.64 (10.77)	
>20	60 (5.00)	70.27 (9.06)	
Monthly income (¥)			
≤5,000	100 (8.30)	63.26 (12.10)	<0.001
5,001–8,000	325 (27.10)	64.58 (11.19)	
8,001–12,000	514 (42.80)	65.47 (10.16)	
>12,000	261 (21.80)	67.64 (10.21)	
Institution			
CDC	70 (5.80)	64.01 (10.78)	0.508
Hospital	523 (43.60)	65.82 (10.76)	
CHS	607 (50.60)	65.42 (10.63)	

¥, Chinese yuan; CDC, center for disease control and prevention; CHS, community health center.

In Table 2, in addition to job rewards, respondents were least satisfied with job rewards and most satisfied with practice environment. In terms of organizational commitment, respondents have the lowest-level opportunity commitment, followed by economic commitment. Reduced personal accomplishment scores the highest among the three dimensions of burnout. Additionally, interpersonal performance and the contextual performance both score lower than task performance.

**Table 2:** Job satisfaction, organizational commitment, burnout and job performance among HCPs.

Dimensions (/total score)	Average score
Job satisfaction (/5)	3.00
Job characteristics	4.21
Practicing environment	2.85
Job rewards	2.61
Interpersonal relationship	3.19
Institutional management	3.10
Organizational commitment (/4)	2.60
Economic commitment	2.50
Affective commitment	2.71
Ideal commitment	2.70
Opportunity commitment	2.22
Normative commitment	2.87
Burnout (/6)	1.86
Emotional exhaustion	1.87
Depersonalization	0.89
Reduced personal accomplishment	2.45
Job performance (/6)	4.46
Task performance	4.54
Interpersonal performance	4.48
Contextual performance	4.33

### Pearson correlation

The Pearson correlation coefficients of the four study variables are presented in Table 3. Job performance was significantly positively correlated with job performance (*r* = 0.25, *p* < 0.01), and organizational commitment (*r* = 0.24, *p* < 0.01), and negatively correlated to burnout (*r* = −0.41, *p* < 0.01). Job satisfaction was significantly positively correlated with organizational commitment (*r* = 0.47, *p* < 0.01) and negatively associated with burnout (*r* = −0.38, *p* < 0.01). There also was significant relationship between organizational commitment and burnout (*r* = −0.23, *p* < 0.01).

**Table 3:** Pearson’s correlation coefficients of key study variables.

**Variables**	**Mean (SD)**	**1**	**2**	**3**	**4**
Job satisfaction	20.90 (3.74)	1			
Organizational commitment	64.98 (9.13)	0.47^***^	1		
Burnout	40.84 (18.81)	−0.38^***^	−0.23^***^	1	
Job performance	65.52 (10.69)	0.25^***^	0.24^***^	−0.41^***^	1

^***^p < 0.001.

### Regression analysis

Linear regression analysis showed association between job performance and other independent variables adjusting for control variables in Table 4. Control variables alone explained 5% of the overall variance in job performance in block 1. In block 2, job satisfaction was positively associated with self-reported job performance (β =0.73, *p* < 0.01) and it explained another 6% of the overall variance in job performance. In block 3, by adding organizational commitment (β =0.15, *p* < 0.01), the explained variance increased slightly (2%). However, burnout explained another 9% of the variance in job performance in block 4. Among the control variable, gender and age were constantly significantly correlated with job performance in four regression models.

**Table 4:** Predicting factors of self-reported job performance.

Independent variables	Block 1 ( β )	Block 2 ( β )	Block 3 ( β )	Block 4 ( β )
Gender (ref: Male)				
Female	−1.93^**^	−1.75^*^	−1.66^*^	−2.17^**^
Age (ref:<30 years old)				
30–39	1.67	1.81^*^	1.76^*^	0.87
40–49	3.20^*^	3.32^*^	3.04^*^	1.68
≥50	4.78^*^	3.85^*^	3.50	1.53
Education (ref: Three-year college or below)				
Bachelor	1.40	1.53	1.73^*^	1.79^*^
Master and above	0.39	1.05	1.59	2.41^*^
Professional title (ref: no title)				
Primary	0.60	1.12	1.59	1.28
Intermediate	0.68	1.44	1.91	1.25
Vice-senior and above	1.08	1.58	2.17	1.20
Years of working (ref: ≤5 years)				
6–10	−0.41	−0.08	−0.28	0.02
11–20	1.37	1.94^*^	1.66	1.68^*^
>20	2.12	3.01^*^	2.52	2.53
Monthly income (ref: ≤¥5,000)				
≤5,000	0.27	0.38	0.19	−0.23
5,001–8,000	0.28	0.09	−0.11	−0.56
8,001–12,000	0.91	0.42	0.02	−0.29
>12,000	0.00	0.00	0.00	0.00
Type of organization (ref: CDC)				
Hospital	2.03	1.99	1.45	1.20
CHC	1.23	1.34	0.96	0.75
Job satisfaction	–	0.73^**^	0.56^**^	0.19
Organizational commitment	–	–	0.15^**^	0.14^**^
Burnout	–	–	–	−0.19^**^
Constant	61.48^**^	45.22^**^	39.17^**^	57.32^**^
*F*	3.91	6.99	7.28	18.12
*R* ^2^	0.05	0.11	0.13	0.22
Δ*R*^2^	–	0.06	0.02	0.09

^*^p < 0.05; ^**^p < 0.01.

### Path analysis

**Figure 1:**
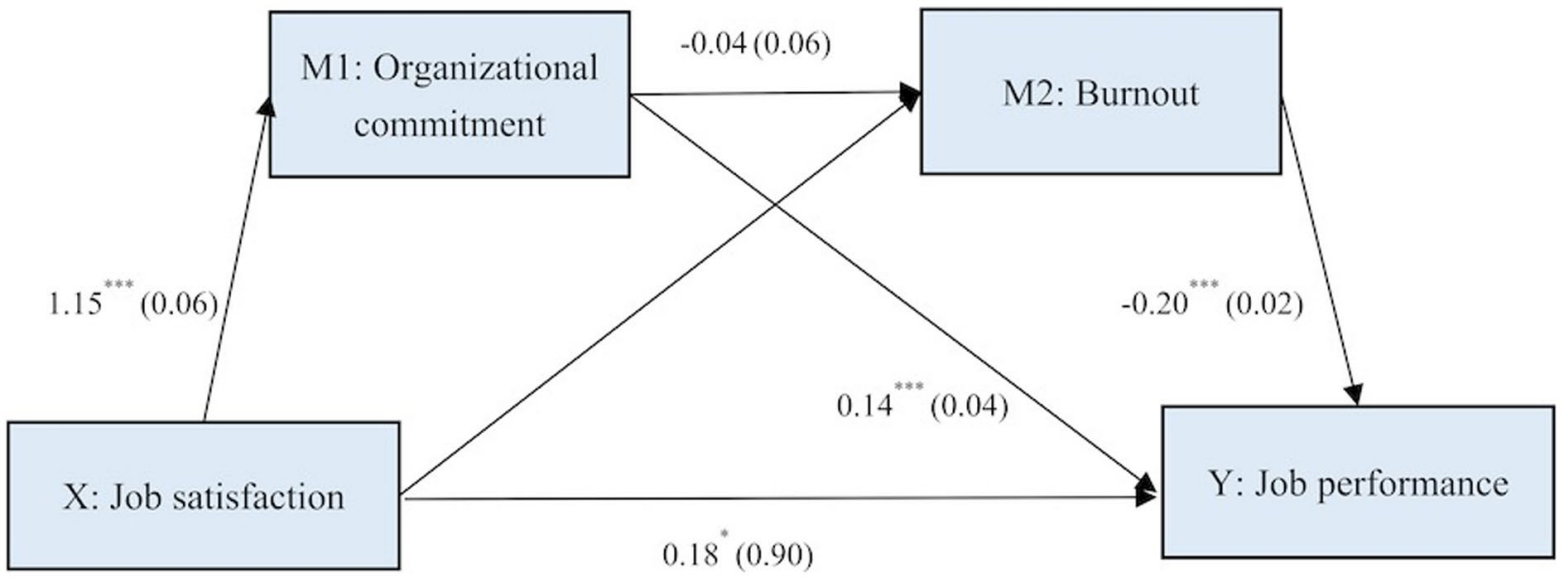
Path diagram of job satisfaction and performance mediated by organizational commitment and burnout with non-standardized β and standard errors. ^*^p < 0.05; ^***^p < 0.001.

Figure 1 shows the coefficients of path from job satisfaction to performance mediated by organizational commitment and burnout adjusting for control variables. The direct effect of job satisfaction on performance was 0.18 (*p* < 0.05). Regarding to the indirect effects, job satisfaction had significant effects on organizational commitment (β =1.15, *p* < 0.001) and burnout (β = − 1.89, *p* < 0.001). Moreover, the effects of organizational commitment and burnout on performance was found to be 0.14 and −0.20 (*p* < 0.001), respectively. However, there was on significant effect (β = − 0.04, *p* > 0.05) of organizational commitment on burnout. Overall, the path diagram showed that parallel multiple mediation had occurred, and organizational commitment and burnout were mediators between job satisfaction and performance.

As shown in Table 5, the total and direct effects of job satisfaction on burnout was 0.73 and 0.18 (*p* < 0.005). The total indirect effect of mediation was 0.55 (*p* < 0.05). The indirect effects of job satisfaction on job performance through organizational commitment alone and burnout alone were 0.17 and 0.37, respectively. The path coefficients of the indirect effects of job satisfaction on performance through both two mediators was 0.01, but it was not significant.

**Table 5:** Direct and direct effects of job satisfaction on job performance.

	Effect	Standard error	Bootstrap 95% CI	
Total effect	0.73	0.08	0.57	0.88
Direct effect	0.18	0.09	0.01	0.36
Total indirect effect	0.55	0.06	0.42	0.66
Indirect effect 1	0.17	0.05	0.06	0.27
Indirect effect 2	0.37	0.05	0.28	0.46
Indirect effect 3	0.01	0.02	−0.03	0.04

Indirect effects 1, Job satisfaction→organizational commitment→Performance; Indirect effects 2, Job satisfaction-→Burnout→Performance; Indirect effects 3, Job satisfaction→organizational commitment→Burnout→Performance.

## Discussion

### Main findings

This study explores the mediating effects of organizational commitment and burnout on job satisfaction and performance among healthcare professionals in a district-level health care system of Shenzhen, China. Results indicate that job satisfaction have significant effects on organizational commitment, burnout and job performance. Moreover, organizational commitment and burnout served as mediators between job satisfaction and job performance.

Comparing with existing studies, this study found the almost same score of organizational commitment with Zhao’s study, higher score of job burnout than in Meng’s study (Zhao et al., 2015a,b). There might be three reasons for the differences. First, the above two studies were conducted in rural township health centers, sharing different background of healthcare system with this study. Second, the establishment of Nanshan Medical Group might have effect on healthcare professionals’ job burnout and organizational commitment. However, we could not estimate the effect without studies before its establishment. Third, the above two studies were conducted before the epidemic of COVID-19, while this study was conducted later. Some studies has demonstrated that COVID-19 has exposed healthcare workers to overwhelming mental pressure, resulting in anxiety, depression and burnout (Francesco et al., 2020; Nicola et al., 2021).

In the linear regression analysis, job satisfaction was positively associated with self-reported job performance and explained another 6% of the overall variance in job performance. Path analysis demonstrated a direct effect of job satisfaction on job performance. Several studies consistently demonstrated the influence of job satisfaction on job performance, supporting the results of the current study (Song, 2014; Zhao et al., 2015b; De Gieter et al., 2018). In additional to direct effects, there were significant indirect effects of job satisfaction on job performance mediated by organizational commitment and burnout. The path coefficient of the indirect effects of job satisfaction on job performance through organizational commitment was as large as the path coefficient of the direct effect of job satisfaction on job performance. Gender, age, years of working and job satisfaction were determinants of organizational commitment. Burnout also serves as mediator between job satisfaction and performance. In contrast to findings from previous studies in rural China, burnout was found to explains most of the variance in job performance in the current study. Wu et al. also found the highest reduced personal accomplishment of healthcare professionals in the CDC, and implied that overworked, absence of financial incentives and promotion opportunities were reasons for the high prevalence of burnout (Wu et al., 2019). Li et al. (2014) found that age, gender and education level were correlated with burnout, which is consistent with this study.

### Implications for health human resources management

The present findings have significant implications for both managers and healthcare professionals in their efforts to improve job satisfaction and performance.

First, female respondents report lower levels of job satisfaction, organizational commitment and job performance than male respondents, indicating that managers in the medical group should pay more attentions to female healthcare professionals. Previous studies in both China and the US also found lower job satisfaction, lower organizational commitment and higher burnout of female physicians than those of male physicians (Jia et al., 2013; Yeluru et al., 2022). And the studies implied that disproportionate burden of household duties, lack of leadership opportunities, lack of support during maternity leave and lactation had tremendous effect on female physicians’ experience with job satisfaction and burnout (Jia et al., 2013; Yeluru et al., 2022). In the past decades, family planning policy in China has undergone gradual adjustment, from one-child policy to two-child policy for couples who were both one-child, to two-child policy for all couples, and to three-child policy for all couples (Zhang et al., 2022). Under the three-child family planning policy, statutory paid maternity leave and fair promotion opportunities might improve job satisfaction and performance and reduce turnover of women workers, especially for female nurses.

Second, satisfaction and organizational commitment of healthcare professionals could be improved by appropriate incentive mechanisms. Rewards scored the lowest among the five dimensions of job satisfaction, and economic commitment scored the second lowest among the five dimensions of organizational commitment, indicating important impacts of economic factors on the job psychology of healthcare professionals. The average monthly income of healthcare professionals of Nanshan District is much higher (twice to five times) than that in other provinces. Increasing incomes through appropriate incentive mechanisms might reduce economic dependence psychologically, such as increasing the proportion of performance-based payments, rewards for over 10 or 20 years work experience in the same institution, and room rent subsidies for new recruits.

Third, it is potential to reduce burnout and improve job performance by improving multidisciplinary healthcare delivery in the medical group. Based on measurement of job satisfaction and performance in this study, satisfaction with interpersonal relationship and interpersonal performance were related to personnel. In the fight against COVID-19, some healthcare professionals in the CDC jointed multidisciplinary family doctor team in the CHSs and they reported improvements in personal accomplishment associated with coordination among healthcare professionals and interaction with patients. Moreover, establishment of the Nanshan Medical Group provided organizational basis for professional and clinical integration. Therefore, it’s necessary for the medical group to promote multidisciplinary healthcare delivery, not only for building integrated high-quality healthcare system but also for improving job performance of healthcare professionals.

### Strengths and limitations

Compared to existing research focusing on job psychology of healthcare professionals in community health centers, tertiary hospital or rural areas, this study targeted healthcare professionals of all health related institutions in the district-level health system, providing comprehensive evidence for health workforce management and related care delivery in the medical group. In terms of data analysis, PROCESS was used for both the mediation analysis but also to control for social-demographic characteristics when examining the association between the four psychological variables. However, there were three limitations of the current study. First, the four job psychology elements of healthcare professionals were assessed by an online questionnaire. The respondents are likely to be younger, with higher education attainment and higher cognitive ability. Based on this study, age had effects on self-reported job performance of the respondents. The influence of selection bias on the results wasn’t estimated in the study. Second, job performance in the study was self-reported. Healthcare professionals who are burnout or unsatisfied with their work may be more likely to underestimate their performance, which might have effect on the results. As the healthcare professionals were from different institutions and different positions, they accomplished different objective performance. Further research will develop a standard assessment tool to assess their subjective performance. Last, the survey was conducted during the COVID-19 pandemic. Although China managed to control COVID-19 in August 2020, the increased work for the preventing and control drained healthcare physicians physically and emotionally (Liu et al., 2020). It might lead to lower job satisfaction, higher burnout level and lower self-reported job performance than before the COVID-19 pandemic (Soto-Rubio et al., 2020).

## Conclusion

This study was a preliminary attempt to examine the relationship of job satisfaction, organizational commitment, burnout and job performance in a district-level health care system of Shenzhen, China. The results indicated that organizational commitment, and burnout serves as mediators of job satisfaction and performance. Burnout explained a larger amount of the variance in job performance than job satisfaction and organizational commitment. Tailored support policies for female healthcare professionals, appropriate incentive mechanisms and improving multidisciplinary healthcare delivery are potential to improve job performance of healthcare professionals in the health care system. Lessons learned from the medical group in Shenzhen may have implications for other district health care systems and other low- and middle-income countries that lack integrated personnel management and high job performance of healthcare professionals in integrated health care systems.

## Data availability statement

The raw data supporting the conclusions of this article will be made available by the authors, without undue reservation.

## Ethics statement

The protocol for the research project has been approved by the Ethics Committee of School of Public Health, Sun Yat-sen University (ref 2020 No. 073). All participants gave written informed consent before recruited into the study.

## Author contributions

YH, FZ, and SB conceptualized this study. XW and YC developed instruments to collect the data. XW and CZ collected the data. CL and CZ analyzed the data. XW wrote the first draft of the manuscript. YH and FZ critically commented the manuscript. All authors have read and approved the final manuscript.

## Funding

This work was supported by the National Social Science Fund of China (grant number 18BGL218) and the National Natural Science Foundation of China (grant number 71804202). The funders had no role in the design of the study and collection, analysis, and interpretation of data, in writing the manuscript and in decision to publish.

## Conflict of interest

YC and FZ are managers of the Shenzhen Nanshan Medical Group.

The remaining authors declare that the research was conducted in the absence of any commercial or financial relationships that could be construed as a potential conflict of interest.

## Publisher’s note

All claims expressed in this article are solely those of the authors and do not necessarily represent those of their affiliated organizations, or those of the publisher, the editors and the reviewers. Any product that may be evaluated in this article, or claim that may be made by its manufacturer, is not guaranteed or endorsed by the publisher.
